# Self-Repair of Rat Cortical Bone Microdamage after Fatigue Loading In Vivo

**DOI:** 10.1155/2013/321074

**Published:** 2013-04-10

**Authors:** Bo Wu, Chan Zhang, Bo Chen, Ling Zhang, Ruchun Dai, Xianping Wu, Yebin Jiang, Eryuan Liao

**Affiliations:** ^1^Institute of Metabolism and Endocrinology, The Second Xiangya Hospital, Central South University, Changsha, Hunan 410011, China; ^2^Department of Radiology, University of Michigan Hospitals, 1500 E Medical Center Drive, Ann Arbor, MI 48109, USA

## Abstract

Bone microdamage can be repaired through bone remodeling induced by loading. In this study, a loading device was developed for improved efficiency and the self-repair process of bone microdamage was studied in ovariectomized rats. First, four-point bending fixtures capable of holding two live rats simultaneously were designed. Rats were loaded and subjected to a sinusoidal wave for 10,000 cycles. They were then divided into four groups to evaluate time points from 1 to 4 weeks in the microdamage repair process. The loaded right ulna was used for microdamage parameter analysis, and the loaded right radius was tested for mechanical properties. In all groups, microdamage consisted primarily of microcracks, which were observed in bone surrounding the force-bearing point. The values of the microdamage parameters were significantly lower at 3 weeks than at 2 weeks. However, none of the differences in mechanical properties between any four groups were statistically significant. This study shows that the improved application of loading in the form of bending for double-rat simultaneous administration was practical and efficient. These results suggest that microdamage was repaired between 2 weeks to 3 weeks after fatigue damage and microdamage is a more sensitive index of bone quality than mechanical properties.

## 1. Introduction

From an engineering materials perspective, fatigue loading conditions can cause the formation and accumulation of microdamage. In bone, microdamage causes a gradual loss of stiffness, which may be considered an indicator of impending failure. Bone develops microdamage after cyclic loading. This triggers remodeling processes in order to maintain skeletal integrity. Animal models have shown that microdamage defects tend to be elliptical in shape tend emerging between osteons, and growing parallel to them [1]. Microcrack parameters can be considered alternative parameters in the evaluation of bone biomechanical quality but diffuse damage cannot [2]. Microcrack length plays an important role in fatigue damage. Microcracks can also be a barrier to microdamage propagation [3]. If the microcracks are <100 *μ*m in length, they may stop at osteonal boundaries, but longer microcracks (length >400 *μ*m) pass through cement lines [4, 5]. Microdamage accumulation is often accompanied by reduced fracture resistance. Three-point bending tests have shown that bone stiffness may drop by 41%, and nanoindentation testing indicated that elastic modulus tissue material properties can be reduced by 26%. The differentials are mainly related to the effects of microcracks [6].

A number of animal microdamage models have used fatigue testing to replicate fatigue damage. Some researchers apply fatigue testing ex vivo to distinguish between different changes in mechanical properties after potential and possible drug therapy. In vivo microdamage models are also used. In these studies, rat ulnae are loaded until 30–40% loss of stiffness is attained [6, 7]. Most models use an axial fatigue loading pattern [7–10]. However, it is difficult to locate the microdamage and force bearing points in these cases. The solution proposed involves using 60% of the total bone length of the ulna or 50% of the humerus [7]. This is not ideal for studies of the relationship between microdamage and stress.

Fatigue loading in vivo has been used to study microdamage-targeted remodeling research. Bone is a living biological material that can repair microdamage and consists of basic multicellular units (BMU). Osteocytes are thought to sense bone damage and mechanical changes induced by intermittent loading and signal to osteoclasts to remove microdamage, and to osteoblasts to form new bone [11]. If the damage is under a certain microdamage threshold, it is repaired by focal bone remodeling. Otherwise, it may lead to a possible fatigue fracture. Microdamage has been shown to trigger the activation of microdamage repair-targeted remodeling [6]. However, linear microcracks, but not diffuse microdamage, drive focal injury induced apoptosis and activates resorption [12]. It has been postulated that mechanical loading may be necessary for microdamage repair and remodeling [13]. Despite extensive work, there has been a limited description of the microdamage repair process induced by intermittent loading of bone. Researchers often apply axial loading, which does not facilitate location of the force-bearing point or the exact position of microdamage. Four-point bending loading fatigue testing has also been conducted in some studies, which circumvents these problems with axial loading. Unfortunately, this type of loading can be time-consuming. The fixtures in these studies allow loading of only one rat at a time. In the present study, we attempt to explain the self-repair process of bone microdamage in ovariectomized (OVX) Sprague-Dawley rats, using an in vivo four-point bending fatigue loading method. Specifically, our research group developed a four-point bending fatigue loading apparatus that can be used on two rats simultaneously. This can produce horizontal fatigue loading and make force-bearing points easy to locate.

## 2. Methods

### 2.1. Animals

All animal procedures were approved by the Institutional Animal Care and Use Committee of the Second Xiangya Hospital of Central South University. Forty Sprague-Dawley rats (female, 7 months old, 350 ± 55 g) were used for this study. The ovariectomy was performed by making an incision on the back to expose the ovaries. The ovaries were clamped and removed with the fallopian tubes being ligated, and the skin was then sutured. Then rats were subjected to loading 3 weeks later after operation using the pressure ended method. Rats were anaesthetized using pentobarbitone injection (2% pentobarbitone, 1.5 mL/Kg) during loading. Before and after loading, the rats were allowed unrestricted cage activity and unlimited access to food and water. At the end of the experiment, animals were euthanized with injection of pentobarbitone into the peritoneal cavity.

### 2.2. Development of Loading Fixture for Two Rats In Vivo

Using the four-point bending principle, we developed a fixture that would allow us to subject two rats to sustained bending fatigue loading simultaneously (China patent number: 201110060409.9). This apparatus is compatible with a PLD-5010 fatigue damage electronic machine (Changchun Research Institute of Test Machines, Changchun, China, Patent number: ZL00225310.0). This machine converts the rotary motion of a motor into rectilinear motion of the mechanical component.

**Figure 1:**
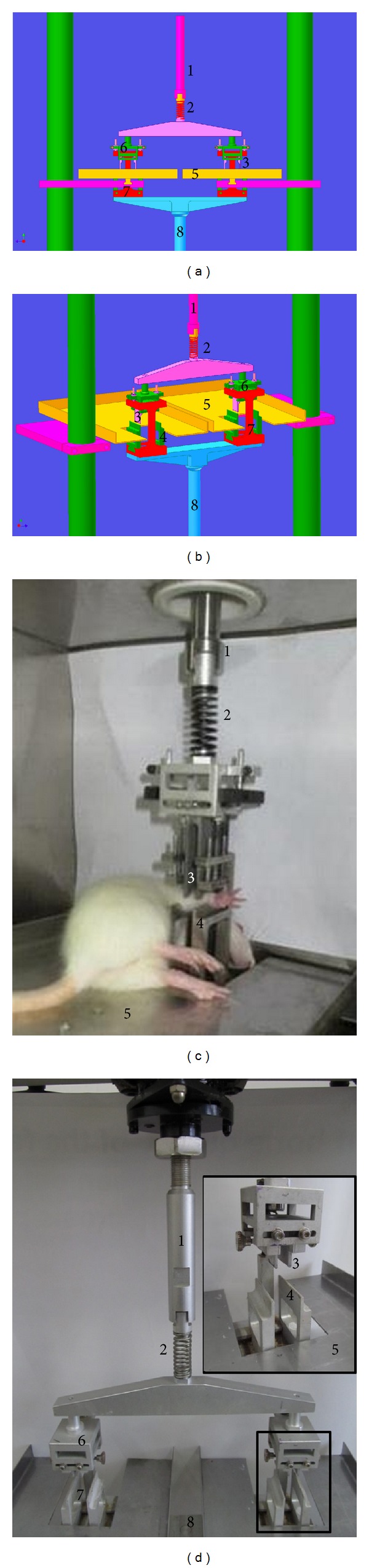
The four-point bending fatigue test apparatus is an open loop system consisting of the following components: (1) upper connecting rod, (2) linear compression spring, (3) upper indenter, (4) lower supports, (5) loading plates where the rat is placed, (6) T-shaped upper indenter slide, (7) C-shaped lower support slide, and (8) lower connecting rod. The picture shows the following. (a) Front view of the apparatus. (b) Lateral view. (c) Fixture with a single rat. (d) Fixture system for two rats.

The fixture was composed of the main loading assembly and loading plates. The anesthetized rats were placed on the loading plates, with room for two rats. The plate position was easily adjusted to accommodate different sizes of rats (Figure 1). The main loading assembly contains the upper connecting rod, linear compression spring, lower connecting rod, upper indenter, and lower supports. The spring was provided with a connecting shaft to the upper connecting rod with a groove connection, facilitating quick and easy disassembly. The maximum load of the spring was 100 N, exceeding the force needed to model the microdamage. This allowed the user to control the load placed on rats' limbs by controlling the deformation and testing force, which were shown in real time on the screen of the measurement system. A T-shaped slide was connected to the upper indenter. This slide contained a rolling bearing in order to reduce the lateral force that may be generated during the testing process and ensure accuracy. The upper indenter could also be moved to accommodate differently sized rats.

This device can produce horizontal fatigue loading and facilitate the precise location of the force bearing point using a mechanical sensor connected to the lower supports, ensuring loading is conducted on the forearms of the rat until a pressure endpoint is reached.

### 2.3. Fatigue Loading In Vivo and Establishment of Self-Repair Model

The rats were anesthetized through intraperitoneal injection of 2% pentobarbitone prior to loading. The right antebrachium of each rat was placed on the lower support bars, and the force bearing point was marked. Cyclic fatigue loading was performed at loads of 0.058 N/g (4 Hz, sinusoidal wave, 10,000 cycles, once every other day for 2 weeks). Each rat's left forearm served as a control. The rats were randomly divided into four groups, sacrificed at 1 week (1st week group), 2 weeks (2nd week group), 3 weeks (3rd week group), and 4 weeks (4th week group 4) after fatigue loading. In order to evaluate bone formation, tetracycline (30 mg/kg, 13 and 14 days before euthanasia) and calcein (5 mg/kg, 3 and 4 days before sacrifice) were given to all rats by intraperitoneal injection.

### 2.4. Basic Fuchsin Staining and Optical Microscopy

After excision of the ulnae, we defined the force-bearing point on the distal ulna as point 1, the point on the proximal ulna as point 4 (upward force), and the middle force-bearing points on the ulna as points 2 and 3 (downward force), and then marked the force-bearing point using thin wire. Ulnae were dehydrated and stained in ascending series of alcohols containing 1% basic fuchsin. They were then subjected to hyalinization and dimethylbenzene and embedded in poly-methylmethacrylate until polymerization was complete. Thick sections (80–100 *μ*m) were then cut transversely and sequentially using a diamond saw. Sections were observed using a Leica DMLA polarized light microscopy (Leica Corporation, Wetzla, Germany). A charge coupled device (CCD) Leica DFC500 (Leica Corporation, Wetzla, Germany) camera was used to capture images of areas where microdamage was found.

Image-analysis software (Leica Qwin image-analysis system, Leica Corporation, Wetzla, Germany) was used to calculate the values of microdamage parameters, such as average microcrack length (Cr.Le), number of microcracks (Cr.N), microcrack surface density (Cr.S.Dn), microcrack density (Cr.Dn), and resorptive space density (Rs.Sp.Dn). These values may indicate the resorptive activity of the remodeling process. Data from each ulna from several sections (4–6/ulna) are summarized [2].

### 2.5. Evaluation of the Mechanical Properties of the Radius

Three-point bending tests to failure were performed under displacement control conditions (electronic universal testing machine WDW3100 (Changchun Research Institute of Test Machines, Changchun, China)). Loading was conducted with the loaded right radius lying on two supports with a span of 12 mm. The cross-head speed was 0.2 mm/s. Matched image analysis software was used to draw the load-deformation curve. Software was used to calculate the linear region between 30% and 50% peak load.

### 2.6. Statistical Analysis

To determine whether fatigue loading caused any decreases in the average length of the microdamage defects, Cr.Le and Cr.S.Dn were averaged and compared to values in the corresponding time groups using independent *t*-tests. The Kruskal-Wallis ANOVA test was used to analyze the loss of mechanical properties, and the Mann-Whitney *U* test was use to analyze Rs.Sp.Dn and Cr.Dn. Significance was set at *P* < 0.05, and data are reported as mean ± standard error of the mean.

## 3. Results and Discussion

### 3.1. Results

**Figure 2:**
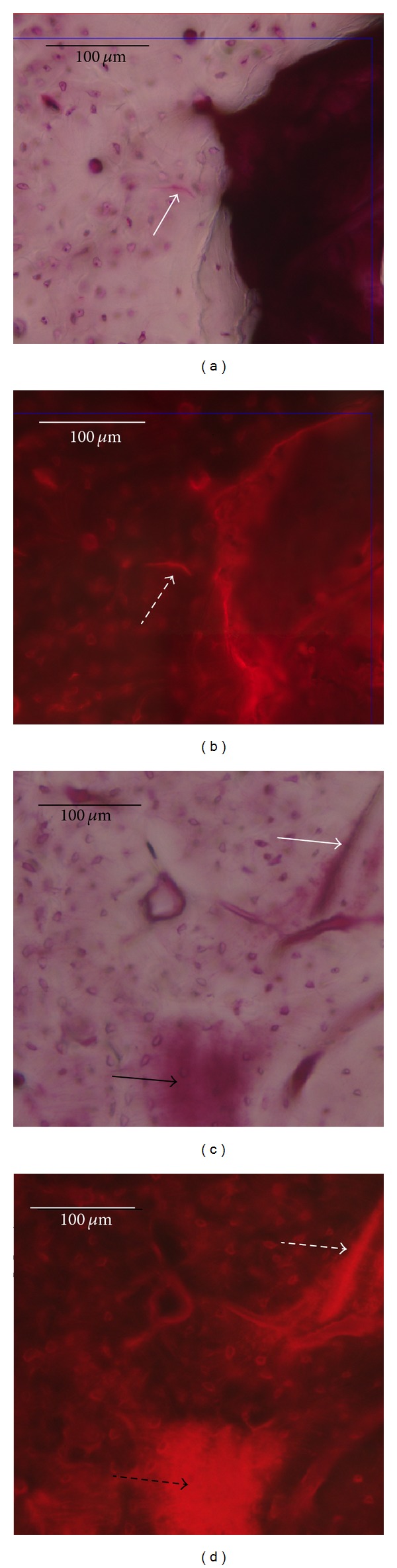
Basic fuchsin staining showing microcracks (white arrow) in (a) OVX 2nd week group rats and (c) OVX 1st week group rats. (b, d) Red epifluorescent light microscopy showing the same microcrack (white dashed arrow) in the identical view. Diffused microdamage (black arrow) was detected in (c) cortical bone (black arrow) and under (d) red epifluorescent light microscopy (black dashed arrow). Scale bars = 100 *μ*m.

Microdamage was observed in all of the loaded ulnae. Microcracks were found to emerge in the region between force-bearing points 1-2 and points 3-4 (on both sides of the bone). Diffuse microdamage was observed in the region between points 2 and 3 (middle of the bone). The greatest amount of microdamage was observed around the force-bearing point, primarily in the form of microcracks (Figure 2). No signs of soft tissue trauma, hematoma, disruption to the periosteum, or intramedullary vasculature were observed.

#### 3.1.1. Microdamage Parameters

**Figure 3:**
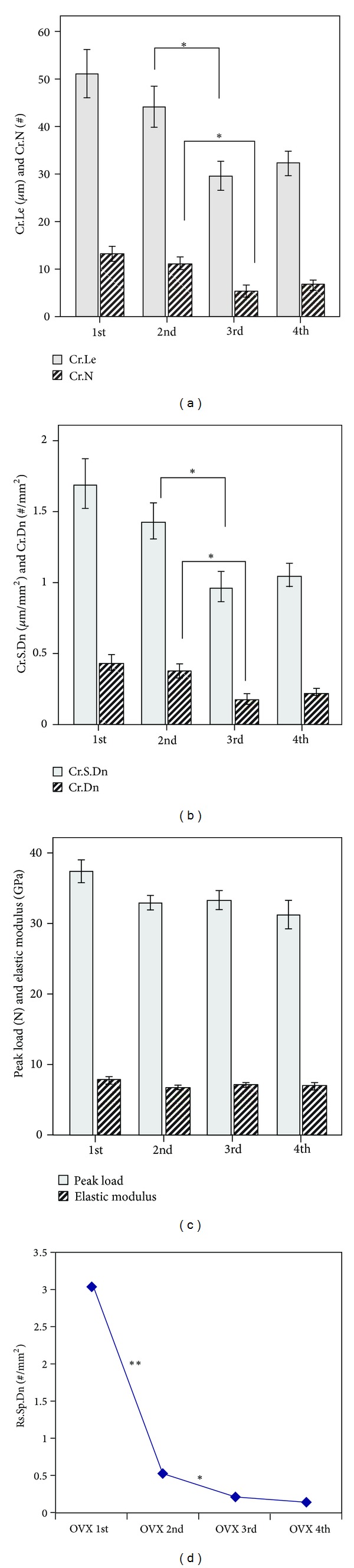
Parameters and mechanical properties of microcracks and absorptive spaces versus time. Specific values are listed in Table 1. (a) Average microcrack length (Cr.Le) and average number of microcracks (Cr.N), (b) microcrack surface density (Cr.S.Dn) and microcrack density (Cr.Dn), (c) peak load and modulus of elasticity, and (d) absorptive lacunar density. *P < 0.05, **P < 0.01.

The values of microdamage parameters, such as average microcrack length (Cr.Le), number of microcracks (Cr.N), microcrack surface density (Cr.S.Dn), and microcrack density (Cr.Dn), were significantly lower in group 2 than in group 3 (*P* < 0.05), but no statistically significant difference was observed between groups 1 and 2 or between groups 3 and 4 (*P* > 0.05) (Figure 3).

#### 3.1.2. Mechanical Properties

Peak loading and elasticity modulus were used as indexes to determine the mechanical properties of the fatigue-loaded radius bones of rats. No statistically significantly differences in peak loading or elasticity modulus were observed among the four groups (*P* > 0.05) (Figure 3, Table 1).

#### 3.1.3. Variations in Absorptive Space

**Figure 4:**
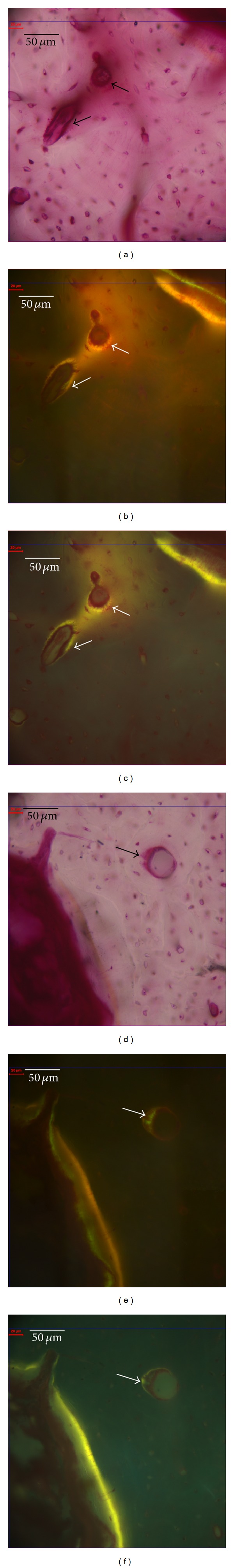
(a, d) Basic fuchsin staining showed resorption spaces (black arrow) in the cortical bone. Blue-violet epifluorescent light microscopy revealed area of osteogenesis (white arrow) and (b, e) green fluorescence attributable to calcein showing osteogenesis during the 4 days preceding euthanasia, (b, e) orange fluorescence and (c, f) yellow fluorescence attributed to acheomycin, which indicated osteogenesis in the 2 weeks before euthanasia. Scale bars = 50 *μ*m.

Absorptive spaces were observed primarily during the first week after fatigue damage. They remained visible during the second and third weeks, but were almost undetectable during the fourth week. The process of osteogenesis can be observed through the two fluorescent lines produced by tetracycline and calcein (Figure 4). The differences in the density of the resorptive spaces (Rs.Sp.Dn) between groups 1 and 2 and between groups 2 and 3 were found to be statistically significant (*P* < 0.05). However, the difference between groups 3 and 4 groups was not statistically significant (*P* > 0.05) (Figure 3, Table 1).

### 3.2. Discussion

To our knowledge, the present study is one of the only studies to develop a fixture capable of loading two rats at a time and identify the window of time during which the self-repair of microdamage takes place after fatigue loading in vivo. Rat ulna bone microdamage was morphologically self-repaired 2-3 weeks after fatigue damage. Microdamage was detected after 4 weeks of fatigue loading. One limitation of this study is that grouping should be added and maintained until microdamage is completely repaired. Microdamage may be a more sensitive index of bone quality than mechanical properties.

Microdamage events are varied, and have different mechanical effects. At least 3 different types of microdamage should be considered. Microcracks and diffuse microdamage have been identified as two kinds of microdamage. Microfractures may or wispy microdamage also be a third, but is not as established. These different forms of damage not only show distinct morphology but are also repaired through different mechanisms. Microcracks and diffuse damage are repaired by damage-targeted remodeling [14]. Linear microcracks can induce self-repair through osteocyte-activated resorption, but diffuse microdamage cannot. This may be due to a lack of apoptotic responses [12]. However, microfractures are repaired in the same manner as normal fractures. This process involves endochondral ossification [15]. Calluses form over fractures in the damaged area. These are eventually remodeled into normal trabecular structures. In the present study, the microdamage detected was mostly microcracks. Other types of damage include diffuse microdamage. No wispy microdamage was found. Microfractures tend to occur only in cancellous bone. We observed only 2 kinds of microdamage in the cortical bones of rats. Loading can cause microdamage, but many studies apply axial fatigue loading, which may limit research meant to explore the relationship between microdamage and stress. Four-point bending loading may address this problem somewhat by allowing the user to more accurately position of the force-bearing point and the microdamage. Some studies have involved bending fatigue loading [16–18]. However, this method requires repeat loading, and existing fixtures can load only one rat at a time, which is inefficient. For this reason, we designed a new type of fixture that can accommodate two rats simultaneously to will improve the efficiency of fatigue testing.

In the bending fatigue test, the force-bearing side experiences pressure load, and the opposite side experiences tensile load with fractures occurring on the tensile side. In our study with four-point bending fatigue testing, microcracks were more common in the pressure side of the bone. This conclusion is similar to the results of another bending fatigue test applied to the long bones of cows [19]. There is an obvious difference between the two loading methods. During axial loading, microdamage, including microcracks and diffused microdamage, were concentrated on ulnar side of the ulna. This may have been because the ulnar side of the bone experienced stronger compressive and tensile force during axial loading. In our study, microcracks emerged on both sides of the bone. Diffused microdamage appeared in the middle of the bone. Distinct categories of force may explain the reason for the diversity observed with respect to microdamage. Shear stress was found to be related to fatigue microcracks and not to diffused microdamage [20]. There was pronounced shear stress between both side of the bone and the areas between two force-bearing points, where one point received upward force while the other point received downward force (point 1 to 2 and point 3 to 4). But no obvious shear stress appeared in the middle of bone (point 2 to 3). This may because it was located between two homodromous pressure points.

To the best of our knowledge, no studies have focused on the time window during which targeted remodeling of microcracks take place. Periosteal woven bone formation can be detected 3–7 days after stress fracture [21]. More pronounced callus formation can be observed 2–4 weeks after osteoporotic fractures. This was demonstrated by callus width and area measurement [22]. Our research indicated that microdamage on rat ulna was mainly repaired between 2-3 weeks of fatigue loading.

It has become clear that microdamage-induced resorption occurs mechanistically via osteocyte apoptosis [14]. Fatigue loading induces microdamage, which may occur in parallel with osteocyte apoptosis and bone resorption [6]. Treatment with apoptotic inhibitors can prevent apoptosis and resorption, with a possible dose-response relationship between the two processes [14]. In the present study, we observed resorption space after one week of fatigue loading. The number of resorption spaces decreased markedly after two weeks of loading. The number of resorption spaces on the ulna was about 6 times greater in cancellous bone than in cortical bone. This may be because the osteoclasts migrate more easily in cancellous bone and because the osteoclast must migrate a larger distance to reach the microdamaged areas in the cortical bone. This also explains why the resorption spaces were more commonly found to localize with regions near the periosteum. It was recently proven that the activation of resorption can be induced by enhanced expression of RANKL [15]. The response may depend on the size of the microcrack. It has been thought that larger cracks may involve greater increases in RANKL and greater decreases in OPG than smaller cracks [23].

Only a few studies have focused on microdamage parameters or considered them as indexes of the curative effects of drugs. Our results indicate that microdamage is a more sensitive index than mechanical properties from our research results. Microdamage parameters may reveal the efficacy of drugs from the microdamage repair field. For example, bisphosphonate may reduce the risk of fracture, but could also impair the repair of microdamage by targeted remodeling. This may be due to oversuppression of bone turnover [24]. Neither estrogen nor raloxifene decreases microcrack surface density or improves mechanical properties of the bone [24, 25]. The effects and limits of many different kinds of drugs may be examined using these techniques.

## 4. Conclusion

In conclusion, we developed a fatigue loading fixture and improved its efficiency. Our data support the conclusion that rat ulna bone microdamage is morphologically self-repaired between week 2 and week 3. Bone microdamage can be visualized and may be a more sensitive index than mechanical properties. Future work based on this research could confirm these results and involve the use of this fixture for the study of bone fatigue loading physiology and for the testing of drug efficacy.

## Figures and Tables

**Table 1:** Microdamage and absorptive space parameters ($\bar{x}\pm s$).

	OVX 1st (*n* = 9)	OVX 2nd (*n* = 9)	OVX 3rd (*n* = 9)	OVX 4th (*n* = 8)
Cr.Le (*μ*m)	51.20 ± 15.17	44.21 ± 13.00^a^	31.53 ± 7.96	29.71 ± 8.98
Cr.S.Dn (*μ*m/mm^2^)	1.70 ± 0.54	1.41 ± 0.300^a^	1.01 ± 0.26	0.96 ± 0.29
Cr.Dn (#/mm^2^)	0.43 ± 0.15	0.36 ± 0.15^a^	0.200 ± 0.12	0.18 ± 0.09
Rs.Sp.Dn (#/mm^2^)	3.032^b^	0.530^a^	0.208	0.143

Values are expressed as means ± SD.

Cr.Le: mean microcrack length; Cr.Dn: microcrack density; Cr.S.Dn: microcrack surface density; Rs.Sp.Dn: absorptive space density.

^a^ *P* < 0.05, relative to with the OVX 3rd week groups; ^b^ *P* < 0.05, relative to the OVX 2nd week group.
